# The Role of Motivation and Commitment in Teachers’ Professional Identity

**DOI:** 10.3389/fpsyg.2022.910747

**Published:** 2022-06-10

**Authors:** Dongmin Ma

**Affiliations:** School of Foreign Studies, North China University of Water Resources and Electric Power, Zhengzhou, China

**Keywords:** multiple factors, commitment, motivation, teacher identity, EFL

## Abstract

Teachers’ professional identity is a feature of an educator that must be planned in an extended, steady, and continuing procedure and typically forms in any particular academic and social setting. As the education profession is largely stressful, it calls for consistent commitment and also motivation to help alleviate the difficulties. Indeed, the educators’ efficiency and effectiveness are mediated by these constructs to both school problems as well as the teaching work as a whole. Besides multiple factors affecting teachers’ professional identity, commitment and motivation have important functions that are addressed by this mini-review of literature. Briefly, several implications are proposed for the EFL instructional recipients.

## Introduction

It is normally maintained that educators are the most significant agents in the learning domain (Pishghadam et al., 2021), and their expert achievement decides to a great extent the final achievement of learners and the academic system overall (Mercer and Dörnyei, 2020). One of the most prominent dimensions of regarding an educator as effective is educator identity, which has attained great interest in academic studies and is recently a rather novel topic (Kalali Sani et al., 2022). Professional identity is characterized as an individual’s professional self-concept dependent upon different elements like experience, conviction, values, motivations, and characteristics. It is an intricate, altering, and individual matter and a continuous cycle and is built by associating with others (Motallebzadeh and Kazemi, 2018). Professional identity, as the center of the teaching career, offers a foundation for self-conception in which every individual discriminates something concerning themselves reliant upon their experiences, reasons, convictions, and values (Derakhshan et al., 2020). It is contended that it is a multidimensional matter involving individual, expert, and surrounding elements that affect educators’ manner in class and their teaching success (Hanna et al., 2020). Professional identity development is among the numerous difficulties EFL educators face all through their primary years of instruction, which has led to considerable conduction of studies (Kanno and Stuart, 2011). Identity definition is a hard challenge because of its several aspects and lively nature which is influenced by lots of elements and modifications through time (Beauchamp and Thomas, 2009). It is said that identity is influenced by internal as well as external factors to the individual (Ruohotie-Lyhty, 2013). It appears to be that mental elements, among many elements that are relevant to educators’ attributes, have the most important function such as commitment, motivation, and self-effectiveness (Kalali Sani et al., 2022). Indeed, for EFL educators, motivation, self-confidence, discernments regarding the work, degree of job fulfillment and further plans are elements that affect their professional identity (Day et al., 2006). Comprehending educators’ identities is crucial to gain a greater depth of insight into the elements that affect educators’ decision-making cycle, demeanors, and convictions (Fogle and Moser, 2017). Accordingly, it can be inferred that identity is an element that affects educators’ motivation, self-effectiveness, commitment, and success.

Commitment is an important problem that is related to educators’ identity referring to the fact that any instructional degree has critical importance and has acquired lots of crucial attention. The management of the academic section calls for a better expert commitment of educators, which is described as an attachment, association, and agreement to the workplace and responsibilities to fulfill the tasks and duties (Zhang et al., 2021). These investigations sometimes address commitment; however, it is a significant dimension of oneself that is sometimes thought to be associated with identity. For instance, turning into an EFL educator calls for the commitment of oneself, not simply fulfilling an assigned task within the class (Kanno and Stuart, 2011). Commitment is also an issue in whether educators work enough to solve the demanding situations of being inexperienced and involved in identity growth and professional learning (Day, 2008). Because education is shown to be a challenging career for educators in due course, their tendency to continue their career is vitally critical to catching up on engagement in education that alludes to commitment. Certainly, educator commitment is highly prioritized because teacher professional identity is grown by educators’ commitment to their careers (Akkerman and Meijer, 2011). In the same vein, a large number of other researchers focused on the connection between teacher professional identity and educator commitment and consider educator commitment as an essential idea regarding professional identity (Day et al., 2006; Cohen, 2010). They accentuated the requirement for standard educator teaching in connection with capability-based and commitment-directed educator teaching. If an educator seeks capabilities and commitment, it will lead to a decent educator presentation. It is presumed that in the functional sense, commitment on the part of teacher educators simply involves both using their best for presenting educator trainees to the capabilities they would require as educators at school and motivating them to teach values of the teaching career.

Furthermore, the last main theory of motivation is the self-determination theory effectively examined relating to educators in the course of their professional path (Ryan and Deci, 2020). The definition of motivation is self-specified and independent, as opposed to externally managed. While educators experience independent and autonomous motivation in their work, they experience positive results, namely, feeling of individual fulfillment, involvement in independent-helping education conducts, improving learners’ independent motivation for learning, and a decrease in the degrees of exhaustion (Roth et al., 2007).

Regarding the function of educators’ professional identity in educators’ fulfillment in instructing and establishing rapport with learners, it is crucial to take into account what may influence such construct. Therefore, based on the review of literature, it could be assumed that within the EFL institutions, there is an inclination toward inspiring educators to enhance their information, education tactics, teaching, and citizenship conduct as some elements of educators’ professional identity. Indeed, teachers experience professional identity change after learning English so their level of commitment toward students should be at the center of attention more, although earlier research has only considered teachers in the perspective of motivation, and self-efficacy in the progress of teachers’ professional identity (Meihami, 2021); however, some factors such as commitment and motivation that might build the professional identity of teachers are ignored. This review makes an effort to fill this lacuna by reviewing those precarious issues such as commitment and motivation in the professional identity of teachers.

## Review of Literature

### Identity

It is believed that identity pertains to human beings’ notion of who they believe they are, and what different human beings think about them (Jonker et al., 2018). Identity as a general term is regarded as a feeling of subjectivity and it will change over time according to the environment where people live and do their jobs (Pennington, 2015). The main identity can offer the capability to start an activity and record an experience and consistent with scholars’ description of identity, Alsup (2006) states that its growth is a continued, always changing event that obliges people to experience digressive anxiety and cognition discordance resulting in elevated comprehension of intersections between people and expert selfhood. Regarding EFL teaching, the issue of educator identity is taken into consideration as an important issue influencing their functioning and therefore focused on by a large number of outstanding scholars (Korthagen, 2003). Establishing and maintaining identity includes the procedure of turning into, which deals with not only our understanding and actions but also our identity; despite this, educational programs mostly run short by concentrating on developing certain expertise and competencies without dealing with the temporary self-evolution (Dall’Alba, 2009). Educators’ PI lies at the center of the education career. It provides educators with a framework to build their very own thoughts of ‘the way of being,’ ‘the way of action,’ and ‘the way of understanding their work and their status in the community. Considerably, educator identity is not constant nor imposed; however, it can be negotiated thru experience and the feeling that is produced by such experience (Sachs, 2005).

### Motivation

Motivation pertains to the reason behind people’s specific decisions, engagement in a task, and persistence in pursuing it and it manages the significance of powerful factors and personal engagement in L2 studying (Ushioda, 2008). It is an individual contrasting element, operating as a motivating factor or inspiration to move or to conduct a thing. It is a passion with a type of thrill that ends in perseverance to get better results, irrespective of their life course, which is associated with stimulating goal-oriented behaviors (Singh, 2011). Two different types of motivation exist: independent and regulated motivation. In one aspect, independent motivation alludes to action with a sense of decisiveness, facing selections and it is related to higher levels of psychological well-being, higher will and decisiveness, higher mental skills, higher levels of job consent, and organizational loyalty and in another aspect, regulated motivation refers to working with a sense of obligation (Fokkens-Bruinsma et al., 2018).

### Commitment

Commitment refers to behavior or psychological state explaining the employee-employer relationship which ultimately affects their tendency to stay or quit the organization (Kotzé and Nel, 2020). Particularly, teacher commitment is fundamental for excellent instructing and it integrates a commitment to the school, learners, vocation persistence, skillful knowledge base, and teaching profession (Crosswell and Elliott, 2004) and teachers’ commitment reflects teachers’ sense of loyalty and dependence on the enterprise they work for and is shown as a substantial indicator of numerous learning and psychological outcomes (Day, 2008). The development of commitment within the academic environment may be logically expected to contain energetic and mutual relationships among various mental, relational, and surrounding factors (Human-Vogel, 2013). Considering such repeated interplay, multiple commitments have higher necessity compared to others in different instances, and also the strength of such commitments depends on the outcomes of various powers in people’s lives (Choi and Tang, 2009). Committed teachers get engaged in communication with their learners and take into account their improvement and they makes an effort for competence in nurturing various methods (Day, 2008).

## Conclusion

Teacher professional identity is central to education that is a rather challenging assignment because it is an important factor in making sure of educator motivation, maintaining the educators’ commitment to their career, and enhancing their philosophy of education (Derakhshan, 2022). In addition, it forms educators’ understanding of their roles, academic reforms, modifications in the educational program, using approaches and techniques, and their association to different important issues inside the academic setting and the way they manage those. Educators’ identity entails the power of commitment and dependence on education activity and/or on education in a particular subject or setting (Pennington, 2015). Education is a difficult and demanding career and therefore, it needs devotion to learners and education activity, which allows commitment to the front. Commitment is an especially associated notion of teacher professional identity as educators need to maintain their intrinsic motivation to meet academic expectations, learner desires, and individual purposes (Pennington, 2015). It can be concluded that commitment is required for educators to get engaged in some tasks and to have a potent teacher professional identity, it is expected that educators keep their commitment to their profession because the level of commitment plays a role in whether or not an educator needs to maintain his education career with pleasure or quit the task with exhaustion and burnout. Consequently, it is claimed that teachers’ professional identity and their commitment degree are interdependent and interrelated (Keskin, 2020).

## Implications and Suggestions for Further Research

This review of literature has some implications for all stakeholders in the educational domain like teachers, school principals, and policymakers because comprehending teacher professional identity refers to comprehending educators’ activities and commitment and motivation for their careers which is central to their measures (Day et al., 2006). Educators must be told of the significance of motivation since by building their enthusiasm, their profession is being formed and this identity decides their work presentation and career success at the workplace. Another suggestion of the current research can be oriented mainly toward teachers and managers in EFL educator and instructor growth organizations to offer more regard to educators’ identity and their motivation and to involve these concepts in the syllabus to prepare and offer preservice teachers with greater educators’ degree of motivation and commitment as well as a deeper comprehension of organizational and professional identities. It is revealed that some features such as motivation and commitment in the teaching occupation are deemed as the most important constructs of EFL teachers’ professional identity profile.

Furthermore, the outcomes of the present research could imply that EFL educators ought to attempt to determine the possible origins of their motivation, commitment, and efficiency and introduce a few feasible techniques that are prone to improve the level of their comprehension of these factors. Results of the current research can also assist teacher educators and syllabus planners with understanding EFL educator features such as professional identity and its connection to academic motivation and commitment more intensely. EFL educators may enhance their education through intentional and analytical focusing on their individual identities within the education procedure. Educators are needed to be committed to a professional information base to catch up with the newest developments in the academic subject. Such sort of commitment consists of a more potent and greater properly-settled professional identity as educators with professional expertise base commitment tend to indicate more attempt to participate in various workshops, meetings, and different professional improvement tasks associated with their field of education to enhance themselves for turning into a more powerful and competent educator.

Professional identity as a section of educators’ identity pertains to the alternative language educators’ individual features like their motivation and their commitment. Also, as the teachers’ professional identity is viewed as the principal of their teaching capabilities that regulates their teaching preparation, motivation, and commitment, effective results are anticipated to be gained from prolonged studies in this domain. Designing educators’ effective identity helps their commitment to learning, increasing their comprehension and skills and encouraging them to play a dynamic role in their education (Izadinia, 2018). More empirical studies can be done to collect qualitative data such as interviews about how teachers perceive their professional identities concerning commitment and motivation. More action research and reflective practice can be done in future to show teachers’ reflection on their classroom performances that can develop teachers’ identity. Also, as other concepts, such as teacher motivation and emotion are related to EFL teachers’ professional identity development, more studies can be done to consider them in the case of eachers’ professional identity progress.

## Author Contributions

The author confirms being the sole contributor of this work and has approved it for publication.

## Conflict of Interest

The author declares that the research was conducted in the absence of any commercial or financial relationships that could be construed as a potential conflict of interest.

## Publisher’s Note

All claims expressed in this article are solely those of the authors and do not necessarily represent those of their affiliated organizations, or those of the publisher, the editors and the reviewers. Any product that may be evaluated in this article, or claim that may be made by its manufacturer, is not guaranteed or endorsed by the publisher.
